# Dynamics of immunoglobulin G subclasses during the first two years of life in Malawian infants born to HIV-positive mothers

**DOI:** 10.1186/s12887-020-02091-z

**Published:** 2020-04-23

**Authors:** Silvia Baroncelli, Clementina Maria Galluzzo, Giuseppe Liotta, Mauro Andreotti, Stefano Orlando, Fausto Ciccacci, Haswell Jere, Richard Luhanga, Jean Baptiste Sagno, Roberta Amici, Maria Cristina Marazzi, Marina Giuliano

**Affiliations:** 1 National Center for Global Health, Istituto Superiore di Sanità, Viale Regina Elena 299, 00161 Rome, Italy; 2 Department of Biomedicine and Preventio, University of Rome Tor Vergata, Rome, Italy; 3 Saint Camillus International University of Health Sciences, Rome, Italy; 4 DREAM Program, Community of S. Egidio, Blantyre, Malawi; 5 Department of Human Sciences, LUMSA University, Rome, Italy

**Keywords:** Immunoglobulin G, Isotypes, Infants, HIV, Malawi

## Abstract

**Background:**

Maternal antibodies are key components of the protective responses of infants who are unable to produce their own IgG until 6 months of life. There is evidence that HIV-exposed uninfected children (HEU) have IgG levels abnormalities, that can be partially responsible for the higher vulnerability to infections in the first 2 years of the life of this population.

This retrospective study aimed to characterize the dynamics in plasma levels of total IgG and their isotypes during the first 2 years of life in HEU infants exclusively breastfed through 6 months of age.

**Methods:**

Total IgG, IgG1, IgG2, IgG3 and IgG4 isotypes, and IgM and IgA plasma concentrations were determined by nephelometric methods in 30 Malawian infants born to HIV-positive women at month 1, 6 and 24 of life.

**Results:**

At 1-month infants had a median concentration of total IgG of 8.48 g/l, (IQR 7.57–9.15), with an overrepresentation of the IgG1 isotype (89.0% of total) and low levels of IgG2 (0.52 g/l, IQR, 0.46–0.65). Total IgG and IgG1 concentrations were lower at 6 months (− 2.1 and − 1.12 g/dl, respectively) reflecting disappearance of maternal antibodies, but at 24 months their levels were higher with respect to the reported reference values for age-matched pairs. Abnormal isotype distribution was still present at 24 months with IgG2 remaining strongly underrepresented (0.87 g/l, 7.5% of total IgG).

**Conclusion:**

HIV exposure during pregnancy and breastfeeding seems to influence the IgG maturation and isotype distribution that persist in 2-year old infants.

## Background

The process of immunoglobulins development and maturation starts during intrauterine life [1] however, the fetus can not produce IgGs, that are received from the mother in a complex mechanism of selective placental passage (preferential transport occurs for the IgG1 isotype followed by IgG4, IgG3 and IgG2 [2]. Neonates are therefore born with a functional immaturity of the immune system and early protection initially relies on the presence of maternal antibodies [3]. Only after the first months of life will infants start to produce their own IgGs, achieving the full immune competence only in late adolescence [4].

In maternal pathological conditions, such as infections and/or inflammatory status the bidirectional fetal-maternal immune cross-talk, including the passage of IgG from mother to fetus, can be altered with important consequences for offspring’s health [5, 6]. Clinical and epidemiological studies reported evidence that maternal HIV infection can deeply affect the maternal/fetal unit, interfering with the immunomodulatory factors which shape immune maturation in fetuses [7, 8]. Immunological abnormalities have been observed in HIV-exposed uninfected (HEU) children, including defects in CD4+ helper T cells and in immune regulatory function [9], and low responsiveness to vaccination [10]. In particular, maternal transplacental transfer of IgGs is inadequate in HIV-exposed children. In healthy pregnancies, full-term neonates have a cord blood IgG concentration often exceeding the maternal plasma concentration [11], but in HIV infection significant reduction of the IgG child/maternal ratio (CMR) has been observed [12]. Several studies have shown that HEU newborns have lower levels of Hib-, pertussis-, pneumococcus-, and tetanus-specific antibodies when compared to non-HIV exposed peers [13]. HIV studies on antenatal vaccine programs have also reported impaired passage through the placenta [14–16].

However, while the decreased transplacental passage has been extensively demonstrated, only a few studies have investigated the subsequent development and maturation of total IgG and IgG isotypes in HEU infants. Immunoglobulins have a key role in the response against pathogens and in the development of adequate responses to vaccinations [17] and the determination of their levels can provide useful information on the status of the humoral immune system. IgGs ranges are well established in adult populations from different geographical areas [18], but the reference intervals are still uncertain in infants since many external factors, such as in utero stimuli, genetic and environmental influences, and exposition to pathogens, could impact on the dynamic process of immunoglobulin development and maturation [2, 11, 19]. Because of the limited number of studies reporting the dynamics of IgG levels in African children, there is a need for a better characterization of the immunoglobulin profile in these populations. The present study is therefore aimed to assess the IgG and IgG subclasses levels during the first 2 years of the life of Malawian infants born to HIV+ mothers.

## Methods

### Study population

The study population included infants enrolled in a cohort study [SMAC (Safe Milk for African Children) study], conducted in Malawi (enrollment: February 2008 – February 2009), and investigating the safety and efficacy of antiretroviral therapy (ART) administration in HIV+ pregnant and lactating women. Study design, clinical details, and antiretroviral strategies have been previously described [20]. The original study did not include a control group. The antiretroviral strategy followed the criteria for treatment in use in Malawi at the time [21]. Naïve HIV-positive women with a CD4+ cell count < 350 CD4 cell/μl started ART as soon as possible after the first trimester, with a combination of stavudine (d4T 30 mg twice daily), lamivudine (3TC, 150 mg twice daily) and nevirapine (200 mg twice daily) and continued the same treatment after the end of breastfeeding. For women with a CD4+ cell count > 350 cell/μl ART was started at 25 weeks of pregnancy with zidovudine (ZDV, 300 mg twice daily), lamivudine and nevirapine, according to the DREAM program [22] and was continued until 6 months after delivery (end of breastfeeding period). All infants received a single dose of NVP syrup (2 mg/kg of body weight) within 72 h of birth.

Gestational age information was self-reported by the women, without obstetric ultrasound confirmation. Therefore this information was not considered reliable. We used neonatal weight as a possible surrogate measure, and according to the World Health Organization (WHO) indication, a neonatal weight < 2500 g was considered low birth weight [23].

Inclusion criteria for this substudy were based on the availability of infant plasma samples at the following timepoints: month 1, month 6 (optional), and month 24 (a total of 30 HEU infants). We have also included 5 additional infants, who had acquired HIV infection and had samples available (in the entire population of 300 infants of the SMAC study a total of 8 infants had acquired the infection).

Since their sample availability was incomplete and the timing of infection was different we decided to analyze them separately.

The study was conducted in Blantyre and Lilongwe, within the DREAM (Drug Resource Enhancement against AIDS and Malnutrition) Program of the Community of S. Egidio, an Italian faith-based non-governmental organization.

### Plasma IgG levels and IgG subclasses

Total IgG and IgG subclass plasma levels were determined using IgG total, IgG1, IgG2, IgG3 and IgG4 reagents (Siemens, Siemens Healthcare Diagnostics) and read by automatized nephelometry (BN ProSpec® System analyzer, Siemens Healthcare Diagnostics). Total IgA and IgM levels were also determined using the same methodology.

Since no IgGs reference intervals were available from healthy infants born in the same geographic/socioeconomic setting of our study, we contextualized our results reporting IgG maturation trend from recent studies on healthy infants from other countries/regions. IgGs reference intervals from other studies were merely used to depict physiological age-specific changes in Igs levels, and no statistical analyses were performed.

### Statistical analysis

The SPSS software version 25 (IBM Corp, 2017, Armonk, NY, USA) was used for statistical analyses. Results are presented as medians with interquartile range (IQR) and percentages. Longitudinal differences were determined using the Wilcoxon Signed Rank Test and Spearman’s correlation coefficient was used for the correlation analysis between quantitative variables. Differences were considered statistically significant when *p* < 0.05.

## Results

### Population characteristics

All infants were delivered vaginally. Their mothers’ characteristics are reported in Table 1. The median age was 28 years, and the median ART duration during pregnancy was 10 weeks. During the study, good adherence to the drug strategy was observed in women, and 6 months postpartum rate of viral suppression (< 400 HIV-RNA copies/ml) was over 90%.

**Table 1:** Maternal and infant characteristics. Values are expressed as medians with interquartile range or percentage

**Mothers**	
N.	30
Age (years)	28.0 (23.8–31.3)
WHO stage I, II, III (%)	70/23.3/6.7
CD4+ cell count (cells/μl)	322 (211–469)
CD4 < 350 cells/μl (n, %)	16 (55.2)
HIV-RNA (log copies/ml)	3.92 (3.07–4.43)
Weeks of ART in pregnancy	10 (5.7–14)
**Infants**	
weight at birth^a^ (kg)	3.35 (3.06–3.53)
low birth weight^b^ n	3
gender (female, n %)	13, 43.3%

^a^Neonatal weight was recorded within 15 days from delivery

^b^The neonatal weight < 2500 g was considered low birth weight [23]

**Fig. 1:**
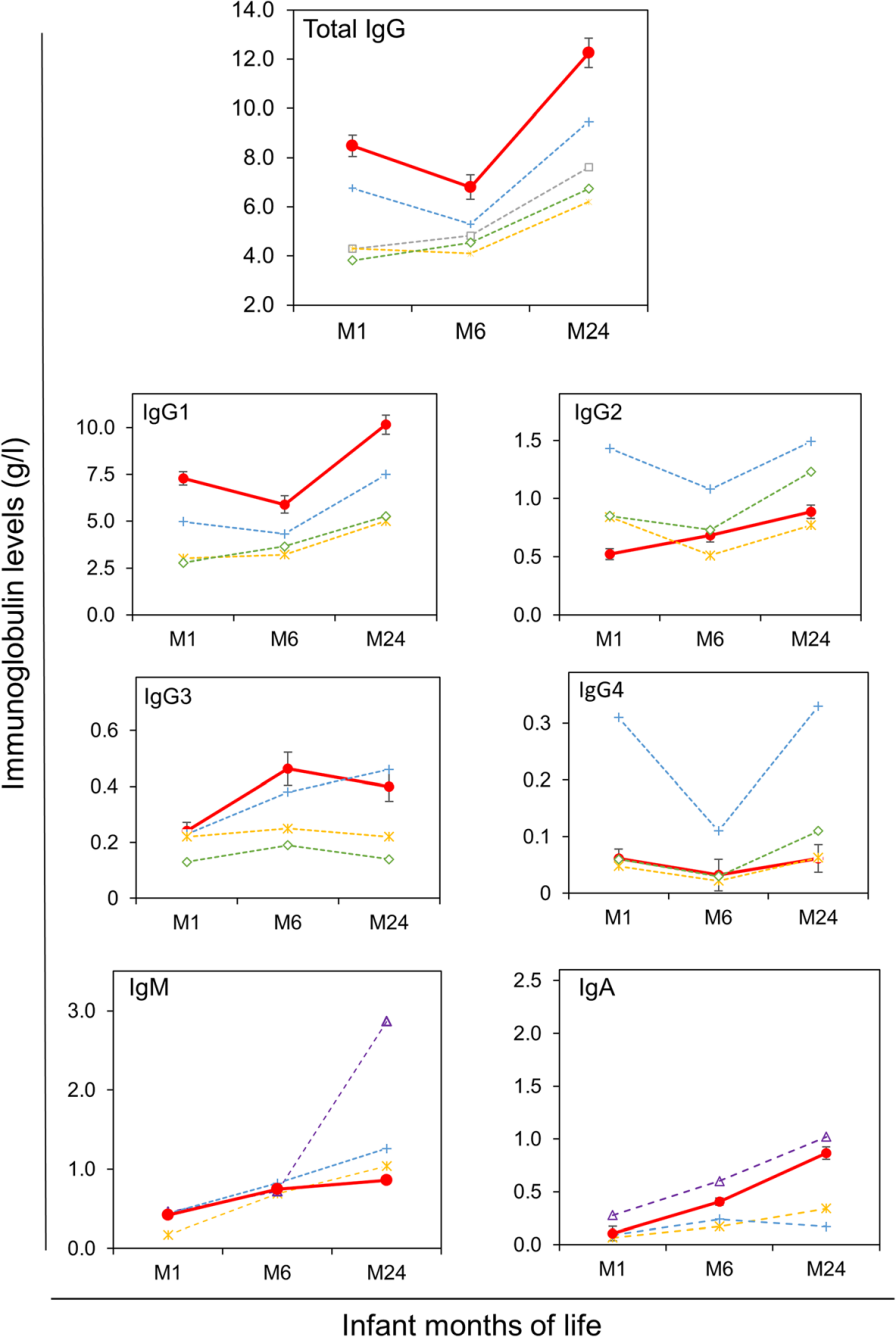
Longitudinal changes of total IgG, subclasses and IgA and IgM. Red lines ad dots  indicate IgG levels of our cohort of HEU Malawian infants. Dotted lines indicate reference values from recent studies on healthy infants of Turkish (grey)  [19], Canadian (yellow)  [24], Thai (blue)  [25] Chinese (green)  [26], and Indian (purple)  [27] origins

Infants were exclusively breastfed for 6 months when all mothers received ART. IgG and IgG isotype levels were determined in 1, 6 and 24-month old infants. The results are reported in Fig. 1 and Table 2.

**Table 2:** Characteristics of 30 HIV-exposed uninfected infants at the time points of the study. Values are expressed in median and interquartile range

	Month 1 (***n*** = 30)	Month 6 (***n*** = 15)	Month 24 (***n*** = 30)
Weight (kg)	4.100 (3.500–4.300)	7.300 (6.405–4.890)	10.0 (9.6–11.2)
Hb (g/dl)	11.3 (10.6–12.1)	10.0 (9.38–10.63)	10.2 (9.55–10.93)
IgG (g/dl)	8.48 (7.57–9.15)	6.80 (6.17–7.78)	12.3 (10.2–13.8)
IgG1 g/l	7.27 (6.65–8.18)	5.59 (5.56–7.25)	10.1 (8.68–11.53)
%	*86.6 (84.1–88.3)*	*88.5 (86.9–91.9)*	*86.7 (84.0–88.1)*
IgG2 g/l	0.52 (0.46–0.65)	0.68 (0.55–0.96)	0.87 (0.68–1.12)
%	*6.65 (5.5–9.03)*	*10.0 (9.0–12.3)*	*7.4 (6.2–9.5)*
IgG3 g/l	0.24 (0.19–0.35)	0.463 (0.32–0.52)	0.400 (0.324–0.602)
%	3.2 (2.4–3.8)	6.01 (4.5–6.9)	3.3 (2.8–4.9)
IgG4 g/l	0.06 (0.03–0.13)	0.032 (0.02–0.05)	0.061 (0.038–0.09)
%	0.84 (0.48–1.38)	0.50 (0.29–0.60)	0.58 (0.36–0.82)
IgGA (g/dl)	0.11 (0.08–0.16)	0.41 (0.32–0.51)	0.87 (0.63–1.00)
IgM (g/dl)	0.42 (0.27–0.52)	0.75 (0.47–0.92)	0.86 (0.75–1.12)

### Immunoglobulins maturation in infants

**Fig. 2:**
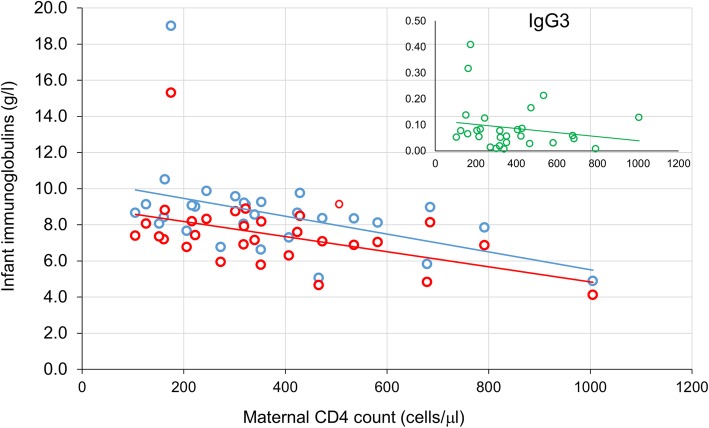
Correlation between maternal CD4+ cell count and immunoglobulins levels in 1-month old HEU infants of the study. Blue circle: total IgG; red circle: IgG1, green circle IgG4

At 1 month the median IgG total level was 8.48 g/l (IQR:7.57–9.15) and the IgG1 isotype level (7.27 g/l, IQR:6.65–8.18) accounted for almost 90% of the total IgG. IgG2 levels (median: 0.52 g/l, 6.6% of total IgG) were below 1.0 g/l in all but one infant. The levels of total IgG were inversely correlated to the maternal CD4+ cell count (*r* = − 0.420, *p* = 0.023); this was true also for the cytophilic isotypes (IgG1: *r* = − 0.429, *p* = 0.020; IgG3: *r* = − 0.447, *p* = 0.015) (Fig. 2). No association with maternal viroimmunological parameters was detected for IgG2 or IgG4. At the following time points (months 6 and 24) no significant association between maternal CD4 cell count and the HEU infants IgGs levels were detected. Neither the duration of maternal ART in pregnancy nor the infant’s birth weight was associated with the IgGs maturation process during the study (data not shown).

At 6 months, at the end of the breastfeeding period, a non significant decline in total IgG (− 2.1 g/l, *p* = 0.191), IgG1 and IgG4 levels (− 1.12 g/l, *p* = 0.496 and − 0.04, *p* = 0.100, respectively) was observed in infants. On the contrary, both IgG2 and IgG3 subclasses significantly increased during the first 6 months of life (IgG2 = + 0.24 g/l, IQR: − 0.06 – 0.46, *p* = 0.027; IgG3 = + 0.054, IQR: 0.09–0.34, *p* = 0.002).

From month 6 to month 24 the total IgG levels significantly increased (median increase of + 4.03 g/l, IQR: 1.01–7.4, *p* = 0.005). A similar trend was observed for IgG1 (+ 3.2 g/l, IQR; 0.7–5.6, *p* = 0.004). IgG2 showed a median increase of 0.19 g/l (*p* = 0.020), while IgG3 levels remained similar to those found at 6 months and IgG4 showed a non-significant increase (*p* = 0.371). The dynamics of IgGs changes are reported in Fig. 1a. In the Figure, the values of the present study are compared to the IgGs values obtained in recent studies on healthy infants of different ethnical origins [19, 21–23]. With respect to the values reported in the literature, HEU infants in our study at one-month of age had a high level of IgG and IgG1 and low levels of IgG2. IgG3 and IgG4 levels were within the ranges reported. Although the longitudinal changes in IgGs seemed to follow the physiological trend observed in healthy populations, at 24 months the total IgG and IgG1 levels in HEU infants of our cohort were higher with respect to the reported values. The ART interruption in mothers with more than 350 CD4^+^ cell/μl count at 6 months postpartum did not impact IgGs development in 24 month- old infants (data not shown).

**Fig. 3:**
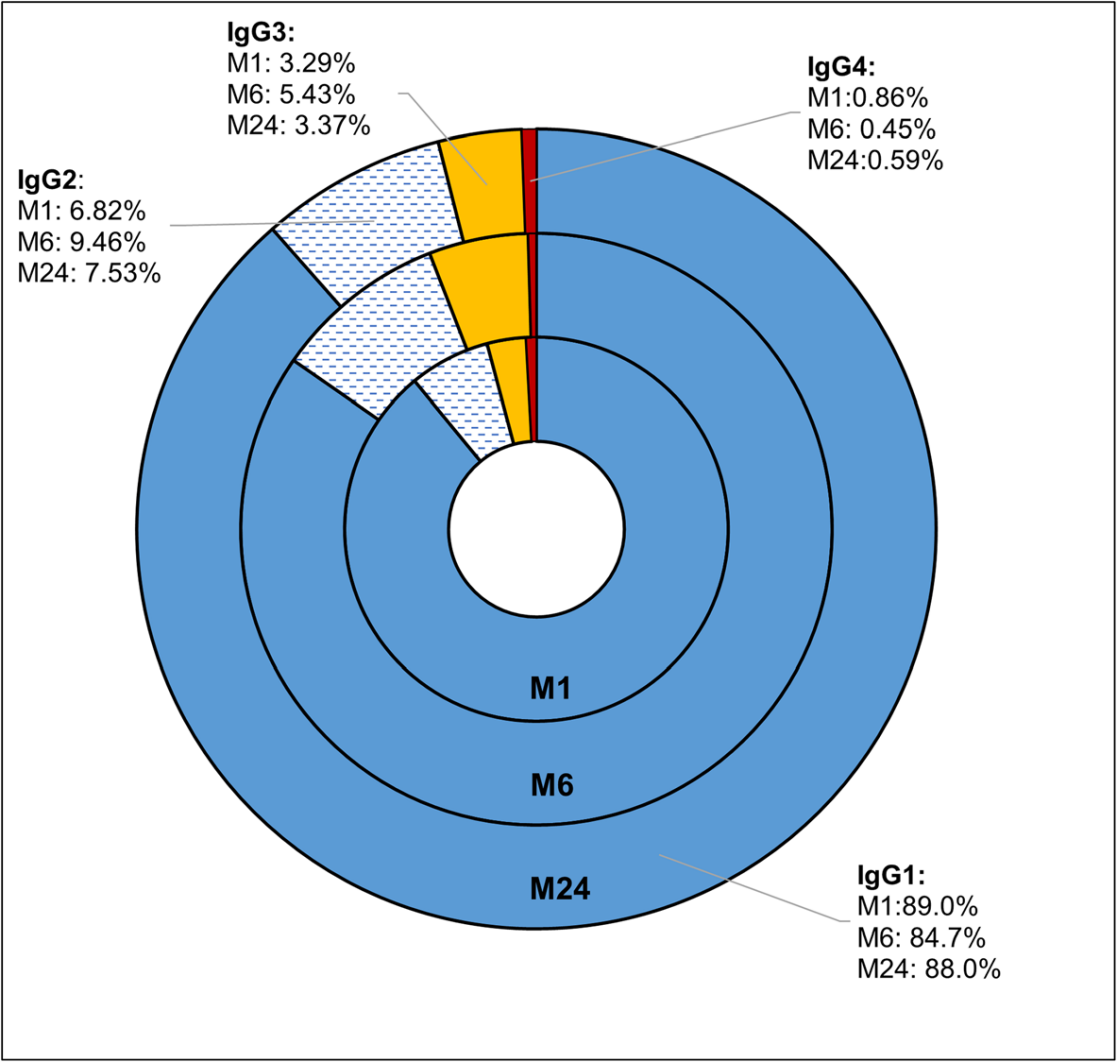
Isotype distribution (percentage of total IgG) in HEU infants during the first 24 months of life

The dynamics of changes in IgG isotype proportions out of the total IgG level are reported in Fig. 3. It can be seen that the proportions of the different isotypes did not change significantly during the course of the study and that at 24 months of age HEU children still have an overrepresentation of IgG1 (more than 80%), with IgG2 accounting for less than 8% of the total IgG.

### IgM and IgA changes during 24 months

IgM and IgA levels were also longitudinally measured in HEU infants. Both increased significantly during the study period (Table 2). In Fig. 1 longitudinal values of IgM and IgA in HEU infants of our cohort in relation to IgM and IgA levels observed in healthy infants of different ethnical origins [19, 22, 24].

### IgG levels in infants infected with HIV during the 24 months

**Fig. 4:**
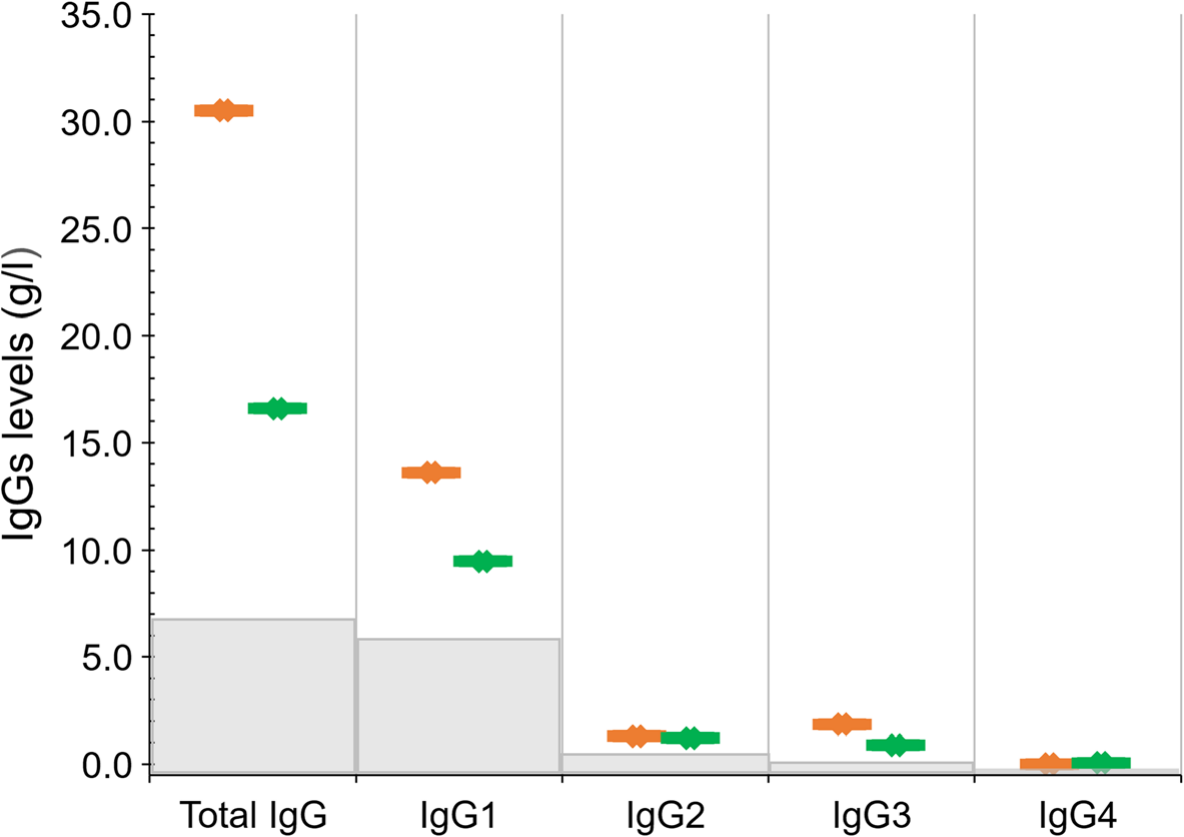
IgGs values at 6 months of the two infants that became HIV infected between 3 and 6 months (orange dot: PT1, green dot: PT2). The light grey boxes represent the median levels of IgGs of their HEU counterparts at the same age

During the SMAC study, only 8 infants acquired HIV infection [20]. In the present study, we could only analyze the incomplete IgG profiles of 5 of them. Two infants (Pt1 and Pt2) acquired the HIV infection between 3 and 6 months. In both cases, IgG levels at 6 months, during the acute phase of the infection, were very high (30.5 and 16.9 g/dl in Pt1 and Pt2, respectively). Moreover, they presented an abnormal distribution of subclasses: IgG1, which in their HEU counterparts represented about 90% of the total IgG, in these two infants accounted for 44.6 and 57.1% of the total IgG, respectively (Fig. 4). Abnormalities in the other subclasses were less evident.

At 24 months samples were available for only 4 HIV+ children. Although under ART, the median level of total IgG in these children was 21.1 g/l (range: 12.8–28.5), 3 out of 4 had hypergammaglobulinemia (total IgG over 15 g/l), and the isotype distribution was similar to those observed in HIV-infected adults: IgG1 = 18.9 g/l, range: 12.2–26.6; IgG2 = 0.97 g/l, range: 0.72–1.24; IgG3 = 0.72 g/l, range: 0.53–1.39; IgG4 = 0.053 g/l, range: 0.03–0.16.

## Discussion

In our cohort, the HIV-exposed uninfected infants were born with high IgG levels with an over-representation of the IgG1 and a low IgG2 concentration. At 2 years of life, the HEU infants still had high levels of total IgG and IgG1 and a substantial disproportion in isotype distribution with respect to reference intervals of same-aged infants. It has to be noted that, to contextualize our findings, the evaluation of IgGs maturation in our cohort was made using reference values from other studies on infants from different ethnic groups.

The longitudinal changes observed in our cohort in IgA and IgM, synthesized by the fetus from early intrauterine life [28], seem to present development within the physiological ranges reported by the literature.

In our study the HEU infants 1-month-old had higher total IgG and IgG1 levels with respect to reference intervals reported in other studies [19, 24–27]; this finding is not surprising since the neonatal IgG profile reflects the maternal IgG levels and distribution. In HIV infection the decline of T-cell functionality is paralleled by the hypergammaglobulinemia and the polarization of immunoglobulins towards IgG1 subclass, indices of B-cells dysregulation [29, 30]. At the same age, however, IgG2 isotype in the HEU infants of our cohort was underrepresented. This finding can be attributable to the lowest affinity of IgG2 isotype with FcRn placental receptors (preferential transport: IgG1 > IgG4 > IgG3 > IgG2) that causes a selective and temporary impairment of IgG2 levels also in healthy neonates [2, 31]. The one-month-old HEU infants of our cohort had a more pronounced deficit with respect to the reference values. Recently, our group reported that the IgG2 deficit in HEU infants could also be attributable to the low levels of circulating IgG2 in HIV-positive pregnant women, suggesting that the low affinity with FcRn receptors of placenta together with maternal low levels could synergically contribute to the IgG2 deficit in neonates [32].

In the first 6 months of life, we observed a temporary decline in total IgG levels, associated with the waning of maternal antibodies, slowly replaced by the HEU infants IgG own production [33]. However, a different pattern was observed for the 4 IgG subclasses: while IgG1 levels showed a decline similar to that of total IgG, IgG2 and IgG3 isotypes increased, and the levels of IgG4 did not change significantly. This finding could be correlated to the role that maternal IgG could have on the neonate’s immune system maturation [34]. Many studies have indeed reported that for several vaccines, such as live attenuated, toxoid and conjugated vaccines, high titers of maternal acquired IgG inhibit the infant’s humoral immune response after infant vaccination [35]. We hypothesize that a similar mechanism could also affect the onset of the infant’s own IgG isotype production.

In our study, neither maternal viroimmunological conditions during pregnancy (CD4 count and ART duration), nor infant’s birth weight was predictive of HEU infants’ IgG maturation over time, but we cannot exclude that the small sample size could have affected the statistical analysis.

The trend we observed in IgGs development during 24 months in the HEU infants of this longitudinal study was not different from those observed in reference intervals reported in the literature; however, at 24 months the level of total IgG was almost double to the reference values, and IgG1 was overexpressed among the subclasses. IgG2 subclass levels at 24 months were slightly lower than the normality range but their proportion over the total IgG levels accounted for 7.4% of total IgG, corresponding to half of the proportion reported for healthy populations (12–19%) [19, 24, 25, 27]. The higher IgG3 levels found in our study with respect to the reference intervals were probably linked to their role in the immunological response to malaria, which is endemic in the area where the study took place [36]. In this view, it has to be considered that the exposition to endemic pathogens is considered one of the major force driving immunological maturation [37]; unfortunately, in our study, the lack of accurate diagnosis during the scheduled medical visits prevented us to determine the real impact of viral, parasitic and bacterial diseases on developing and maturation of immunoglobulin in these children.

Although this study was designed to determine the dynamics of immunoglobulins in HEU children, we had the opportunity to test the IgG levels in infants infected by HIV during breastfeeding. Two children were infected before the physiological initiation of IgG synthesis, and in both cases, at 6 months we found a significant hypergammaglobulinemia, with values similar to those found in HIV+ symptomatic adults [38], and a strong discrepancy between the total IgG levels and the sum of subclasses levels. The nephelometric analysis revealed for both infants that the sum of the subclass measurements accounted for only 41% of the total IgG, with a partial reduction of IgG1 subclass. The result was confirmed in both cases in a repeat test. Although we cannot rule out a technical problem (i.e. reduced interaction of IgG1 protein with the IgG1 antiserum), the finding could indicate a virus interference with the production of IgG subclasses by B cells in acute infection, determining an early impairment of the B cell compartment. The finding in our study could be considered an anecdotal observation however, this topic could be investigated in larger cohorts.

The major limitation of this study is the lack of an appropriate control group (i.e. age-matched children from the same geographical area) which does not allow us to draw definite conclusions on the levels that we found. The availability of reference values in Sub-Saharan African countries remains a challenge due to many factors, including organization aspects and lack of laboratory facilities. We are aware that the use of reference values from populations of different ethnic origin and exposed to different environmental factors, can be problematic [39], but we tried to minimize the problem using reference data selected from very recent studies, in which immunoglobulin determinations were performed using the nephelometric method, which is considered the gold standard for serum protein detection with a high standard of inter- and intra- laboratory precision and reproducibility.

The low number of subjects included is another limitation of this study, although we determined the intra-individual variation over time and observed very low inter-individual variability, that did allow us to be confident with the validity of our results.

## Conclusions

Here we reported the dynamics of IgG development and maturation in HEU infants, suggesting that their IgG profile at 24 months may still present anomalies (mainly represented by hypergammaglobulinemia and low IgG2 levels) probably as a consequence of the early-life exposure to maternal HIV-related immune alterations.

In our study, performed before the adoption of the Option B+ strategy, we found a significant association between a low maternal CD4+ cell count and the levels of IgG in neonates, confirming the impact that compromised viro-immunological maternal conditions could have on the transplacental passage of IgG in neonates [2, 14, 40]. The women of this study received only a median of 10 weeks of ART during pregnancy, a duration that was inadequate to restore the HIV-related maternal immunological dysfunctions which can deeply impact neonates’ health. Future studies will determine if these anomalies in infants may be corrected by the adoption of the current strategy of universal and life-long antiretroviral therapy administration.

## Data Availability

Dataset of the current study is available on reasonable requests.

## Abbreviations

HIV: Human immunodeficiency virus
HEU: HIV exposed uninfected
Ig: Immunoglobulin
CMR: Child/maternal ratio
